# Comparative analysis reveals the Genomic Islands in *Pasteurella multocida* population genetics: on Symbiosis and adaptability

**DOI:** 10.1186/s12864-018-5366-6

**Published:** 2019-01-18

**Authors:** Dekang Zhu, Jiao He, Zhishuang Yang, Mingshu Wang, Renyong Jia, Shun Chen, Mafeng Liu, Xinxin Zhao, Qiao Yang, Ying Wu, Shaqiu Zhang, Yunya Liu, Ling Zhang, Yanling Yu, Yu You, Xiaoyue Chen, Anchun Cheng

**Affiliations:** 1 Research Center of Avian Diseases, College of Veterinary Medicine, Sichuan Agricultural University, Chengdu, Sichuan China; 2 Institute of Preventive Veterinary Medicine, Sichuan Agricultural University, Chengdu, Sichuan China; 3 Key Laboratory of Animal Disease and Human Health of Sichuan Province, Chengdu, Sichuan China

**Keywords:** *Pasteurella multocida*, Genomic islands, Population genetics, Pan-genome, Symbiosis, Adaptability

## Abstract

**Background:**

*Pasteurella multocida* (*P. multocida*) is a widespread opportunistic pathogen that infects human and various animals. Genomic Islands (GIs) are one of the most important mobile components that quickly help bacteria acquire large fragments of foreign genes. However, the effects of GIs on *P. multocida* are unknown in the evolution of bacterial populations.

**Results:**

Ten avian-sourced *P. multocida* obtained through high-throughput sequencing together with 104 publicly available *P. multocida* genomes were used to analyse their population genetics, thus constructed a pan-genome containing 3948 protein-coding genes. Through the pan-genome, the open evolutionary pattern of *P. multocida* was revealed, and the functional components of 944 core genes, 2439 accessory genes and 565 unique genes were analysed. In addition, a total of 280 GIs were predicted in all strains. Combined with the pan-genome of *P. multocida*, the GIs accounted for 5.8% of the core genes in the pan-genome, mainly related to functional metabolic activities; the accessory genes accounted for 42.3%, mainly for the enrichment of adaptive genes; and the unique genes accounted for 35.4%, containing some defence mechanism-related genes.

**Conclusions:**

The effects of GIs on the population genetics of *P. multocida* evolution and adaptation to the environment are reflected by the proportion and function of the pan-genome acquired from GIs, and the large quantities of GI data will aid in additional population genetics studies.

**Electronic supplementary material:**

The online version of this article (10.1186/s12864-018-5366-6) contains supplementary material, which is available to authorized users.

## Background

As the technology-developing capacity and the scale of genomic data developes, population genetics and molecular evolution have become central analytical disciplines [1–3]. The question of how genetic structure and variation in species affect selective forces and adapt to novel conditions has been foundational to the field of population genetics [4, 5]. Gene islands (GIs) are an integrative mobile element, containing large genomic regions (> 10 kb) acquired through horizontal gene transfer (HGT) between different hosts, that might increase the versatility and adaptability of the recipient and enable certain bacteria to occupy entirely new niches. GIs act on for population genetics by facilitating gene exchange within or among species [6, 7]. Lysogenic bacteriophages and plasmids are important enablers of gene flow [8, 9]. They promote the formation of GIs and confer superior mobility as removable genetic components that move through site-specific recombination and integrating into the corresponding chromosomes [10]. Integrative and conjugative elements (ICEs) are a special type of GIs that promote the mobilization of degenerative, immobile GIs and contributing to lateral gene flow [11, 12]. In general, GIs are divided into pathogenicity, resistance, xenobiotic-degradation, metabolic, symbiosis or fitness islands by the environmental context of the bacterium and the one or more important functional genes among them [13, 14].

*Pasteurella multocida* (*P. multocida*) is a causative agent of economically crucial diseases spanning the entire world and is a facultative pathogen of Gram-negative, atrichous, nonspore-bearing but encapsulated coccobacillus [15]. Germs are the sole pathogenic agent that cause pasteurellosis in diversified animals, including atrophic rhinitis (AR) and swine plague hogs, fowl cholera (FC), haemorrhagic septicaemia (HS) of bovines, infectious pneumonia in sheep and snuffles in rabbits [16]. Oliveira Filho (2018) demonstrated that *P. multocida* is able to cause pneumonia in pig without other pathogen (bacteria /virus) [17]. And scratches or bites from infected dogs or cats can sporadically cause human infection [18]. Currently, there have been studies on the virulence genes of *P. multocida* [19], with prospective analysis including forward selection genes and host-virulence interaction genes in the pan-genome [20, 21], and HS-specific genes involving disease-specific diagnostic tests and possible resistance genes [16, 22]. Moreover, there are also a few reports of *P. multocida* GIs, the resistant island ICE*Pmu1* from strain 36,950 [23], and the resistant islands from strains TX1 and BUKK, which are similar to ICE*Pmu1* [16].

However, the extremely dynamic pan-genome of facultative pathogens is largely derived from mobile elements and phage-derived GIs, representing for some taxa the major evolutionary route of facultative pathogens [9, 24]. The close correlation between GIs and bacteriophages and the characteristics of GIs’ large mobile components require more attention. For the facultative pathogens of *P. multocida*, the GI proportions and effects on *P. multocida* population genetics were explored through comparative genomics between the pan-genome and GIs in this article.

## Results

### Genomic characteristic of *P. multocida*

The quantitative data of 10 sequenced avian-sourced strains and 104 reported *P. multocida* strains, 17 complete and 97 draft, are shown in Additional file 1: Table S1. Apart from containing gap aligned areas, the average genome length of the donors was 2,323,144 bp, ranging from 2.20 to 2.70 Mbp. The 114 strains were from 12 different countries. Of all the strains, 18 strains were isolated from poultry, 66 from bovines, 17 from rabbits, 5 from swine, 2 from goats, 1 from canis, 1 from alpaca and 4 from humans. The quantity of the encoded proteins ranged from 1903 (Razi 0002, avian) to 2313 (FDAARGOS_261, human); the average GC content was 40.29%, while the minimum was 36.9% (HN141013, DY120818, avian; CIRMBP-0760, *Oryctolagus cuniculus*) and the maximum was 40.7% (P1062, HS SKN01; cattle); the amount of rRNA ranged from 1 (2000, cattle) to 19 (the great majority), and tRNA ranged from 16 (PmP1933, cattle) to 59 (PmOH1905, canis; Razi_Pm0001, cattle); the pseudogenes ranged in magnitude disparately from 6 (Pm70, avian) to 210 (1500E, avian) (Additional file 2: Table S2). Simultaneously, the scanty rest was interrogative and unfilled portions were uncharted in the database.

### The pan-genome analysis across all strains

**Fig. 1:**
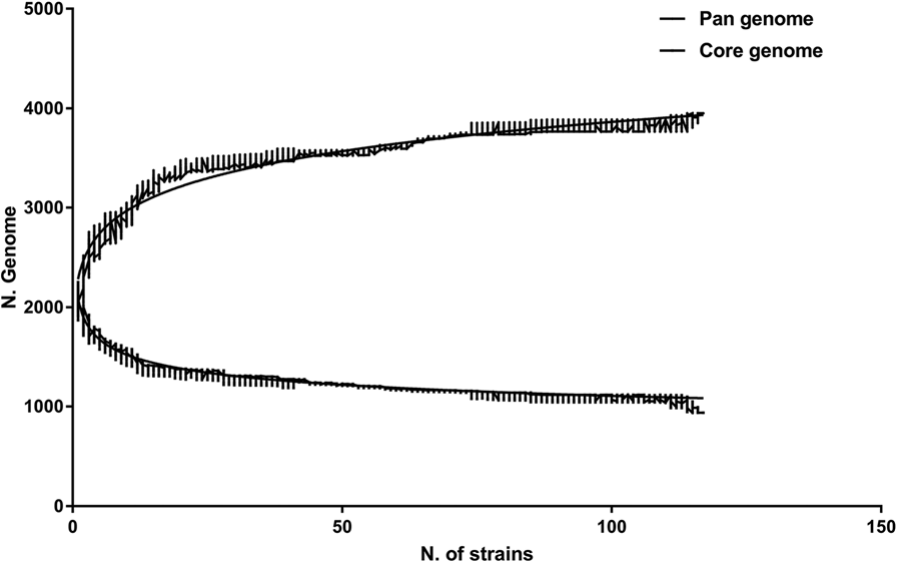
Conserved core genes and the pan-genome for 114 *P. multocida*. The sizes of the pan-genome and core genes are plotted as a function of the number (n) of strains sequentially added. On the above curve, increasing number of pan-genomes with an extrapolated curve increases the number of genes, showing an open pan-genome of *P. multocida*. On the blew curve, the core genes show the exponential decay model based on the median value for conserved genes when increasing numbers of genomes are compared

**Fig. 2:**
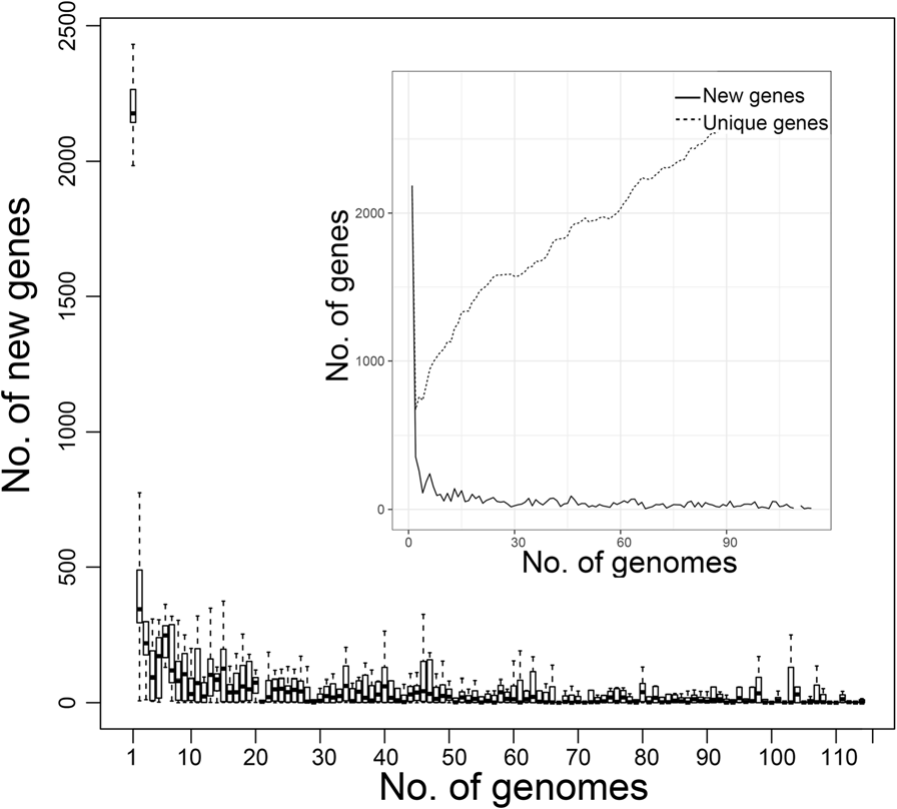
New and unique genes found in *P. multocida* according to the finite supragenome model. The number of new genes and unique genes as a function of the number of sequenced genomes, respectively. The predicted number of new genes approaches zero. The number of unique genes converges to increase progressively

To understand the population genetic information of *P. multocida*, a comparison of the 114 isolates with each other discerned 3948 COGs (Cluster of orthologous genes) in the pan-genome. The ratio of core/accessory/unique genes in each strains was shown in Additional file 3: Figure S1. The core set of the pan-genome was at a rate of 23.9% (944 COGs), and the dispensable genes was 76.1% (3004 COGs). The evolutionary pattern of *P. multocida* was investigated based on 114 isolates (Fig. 1). The mathematical model heap’s law was n_pan_ = 1952.72*N^0.13^. According to 0 < γ < 1 and Fig.1, the pan-genome is still open [25]. The new genes of *P. multocida* fluctuated slightly with the increase of the strain; most of the cases were close to zero, and the unique genes increased in proportion with the increase of strains, and thus, the ability of the strain to obtain foreign elements was stable (Fig. 2).

**Fig. 3:**
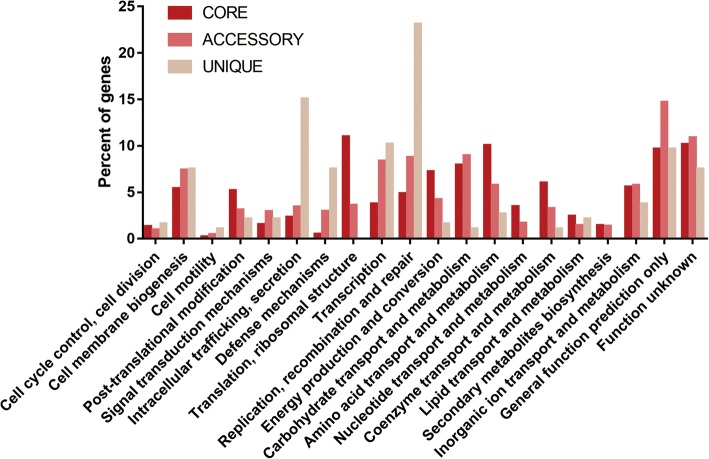
Characterization of the gene sets of all strains showing diverse function. The distribution of COG categories across the core, accessory and unique genome. Dissimilar gene sets are expressed in different colours, with deep red representing the core genes, pale red representing the accessory genes, and dark salmon representing the unique genes. The height of each column rectangle represents the proportion of the functional categories

A functional clustering of the pan-genome was performed with COG (Fig. 3). Apart from the proteins of unknown function or general function only, the main functional categories of all the strains were growth, metabolism, and functional repair; the core genes were more relevant for growth and metabolism activities, such as translation, ribosomal structure and biogenesis; the accessory genes, 61.8% (2439/3948) of the pan-genome, were more relevant for information storage, processing and metabolism, such as carbohydrate transport and metabolism and cell wall or cell membrane biosynthesis. The remaining 565 unique genes, 14.3% (565/3948) of pan-genome, were detected in 40 out of 114 strains, and the results from the specific strains and quantities are shown in Additional file 4: Table S3. According to the functional clustering, the unique genes were mainly related to cellular processes and signalling, mostly encoding for replication, repair, membrane transport, metabolism of cofactors and vitamins, and metabolism of terpenoids and polyketides. *P. multocida* Anand1_poultry contained the largest number of contigs in all strains, with 98 unique genes which were the largest in number compared to other strains. In addition, FDAAROGS_261 and HS_SKN01 also contained 70 and 87 unique genes, respectively, which were significantly higher than those of other strains.

### Alignment and phylogenetic analysis of 114 strains

**Fig. 4:**
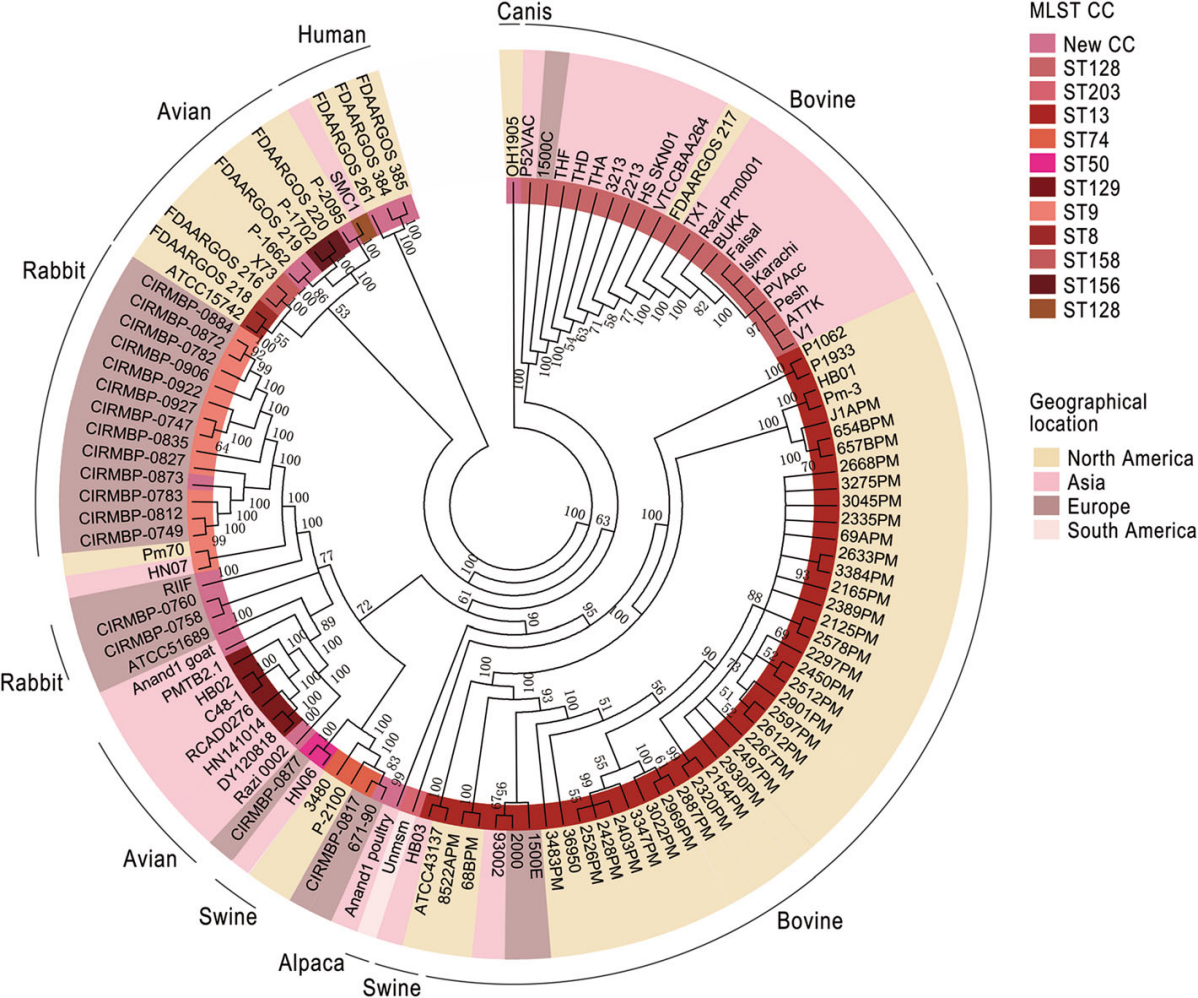
The phylogenetic tree of 114 *P. multocida* based on the core genes. From the inside, the inner circle 1 marks the MLST CC (clonal complexes) corresponding to each strain, and the corresponding sequence type and colour are displayed on the right in the order in which they appear. Circle 2 shows the different geographical locations of the strain in the key colours on the right side, including North America (America and Canada), Asia (China, Thailand, India, Sri Lanka, Pakistan, Malaysia, and Iran), Europe (UK and France), South America (Peru) and unknown geographic sources (Table S1). Circle 3 shows the host source of the isolates

Based on the core genes, the phylogenetic tree was constructed with MEGA software. This tree can be used to understand the relationship between classification and phenotype of strains based on gene levels. The hosts’ distinguishing characteristics were determined according to the separation site and the sequence classification in the phylogenetic tree (Fig. 4). Comparing the results of the core genes with the MLST (Additional file 5: Figure S2), the classification results and the STs of these strains were consistent, meaning they were in the same branch or extremely adjacent branches, as labelled with the same colour in the diagram (Fig. 4). Furthermore, the phylogenetic relationship of these strains also was intimately related to the host. Among them, most strains isolated from the same host type were on the same major branch, distinct from other strains. As shown in the figure, 66 strains from bovine showed a close phylogenetic relationship, excluding 671/90, TB2.1 and ATCC 51689, and divided into 2 groups according to their continent of origin. Eighteen strains from avian sources were mainly distributed into 2 large branches according to geographic location. Among them, the avian-sourced Pm70, pig-sourced HN07, and sheep-derived RIIF were clustered together with 13 rabbit-sourced strains on one large branch; P-2100 clustered with other ST74 strains, including swine-sourced 3480 and rabbit-sourced CIRMBP-0817; Anand1_poultry was located alone on one branch. Only 4 out of 17 rabbit-sourced strains were *P. multocida Subsp. multocida* and non-ST9 type on the outside, and 13 ST9 were *P. multocida subsp. septica* and clustered onto one branch. Two ST13, ATCC 43137 and HB03 of the 5 swine-sourced were aggregated, and 1 ST74, 3480, and 1 ST50, HN06, were clustered. The remaining HN07 was scattered in other regions. Three of the 4 human-sourced strains were significantly different from all other strains and clustered into one group, while SMC1 was close but not in the same large branch. Although the 2 sheep-sourced strains, RIIF and Anand1_goat, did not cluster tightly, they were located in a large branch as shown in Fig. 4. One alpaca-derived strain was separated from the other strains separately, and 1 strain of canis-derived strain was also clustered into a single group. The classification of *P. multocida* on the phylogenetic relationship correlated to the host and geographic location.

### Genomic islands in *P. multocida*

GI regions of all strains were predicted and screened. Precise positioning and functional comments have been applied to parse the complete genomes of 17 *P. multocida*, shown in Additional file 6: File S1. The exact GI location was determined for 17 complete genome sequences according to the search for direct repeat GI sequences and whether they contained the basic GI origin DNA, such as hypothetical protein, phage-related protein, and integrase; the integrase type and integration site were counted and collated (Additional file 7: Table S4). On average, complete genomes from *P. multocida* strains harbor 36 GIs. In the results, there were 2 RGI, one of which contained a large number of integrative conjugative elements (36,950, ICEPmul), 23 PAIs, 3 metabolic islands, 4 symbiosis islands, together with 1 pathogenicity and symbiosis island, 2 pathogenicity and metabolic islands, and 1 pathogenicity and resistance island. The GI integrases in *P. multocida* mostly belong to the reorganization of tyrosine enzyme/integrase families, while a few belong to the Rve family. Ninety-seven draft genomes have been analysed with a similar method using *P. multocida* ATCC 43137 as reference. The regions of GIs without precise starting point were predicted and the interrupted GI fragments were discarded totally because of the limitations of the strains sequence themselves. Hence, 280 GIs were taken, 123 of which were PAIs, 27 RGIs, 97 symbiosis islands, 4 metabolic islands, 7 pathogenicity and metabolic islands, 1 pathogenicity and symbiosis island and 21 pathogenicity and resistance islands (Additional file 7: Table S4 and Additional file 8: Table S5).

Consequently, there were 280 GIs in this experiment. The virulence-related islands accounted for 44% (123 in total) of the all predicted results if the multifunctional GIs were excluded (the above statistical results that were not part of the data of PAIs), or 54% (152 in total) if the multifunctional GIs were included. In addition, the symbiosis islands also occupied a large proportion at 37% (97) or 35% (98). ICE accounted for 11% (32) of the total.

### Prophage regions in *P. multocida*

Temperate phages are important factors in bacterial evolution and the formation of new pathogens and sometimes can be converted into virulent phage to kill bacteria [26–28]. The prophages of 114 *P. multocida* strains were predicted. The prophage-related elements were analysed and 324 phages were identified and described with their general characteristics, including 153 intact bacteriophages, 36 questionable bacteriophages and the 133 incomplete (Additional file 9: File S2 and Additional file 10: Table S7). Only the bacteriophage region in the genome of 93,002 is not present in all strains. According to the formation mechanism of GI, the phage is likely the precursor to the formation of GIs; that is, the phage could lose self-replicating genes and large segments of HGT by integrase, transposase, recombinase and so on between the strains in the form of GIs [6]. Due to the differences in software rearrangement in the prediction of the draft genome strains, only the GI regions of the 17 complete genome strains were compared with their predicted prophage area, and 63.3% (31) of the possible phage regions were found to be approximately the same as those of the GIs (Additional file 10: Table S7). Of these, 64.5% (20) were intact, 22.6% (7) potential and 12.9% (4) incomplete prophage regions.

### The particular correlation of the population genome and GIs

**Fig. 5:**
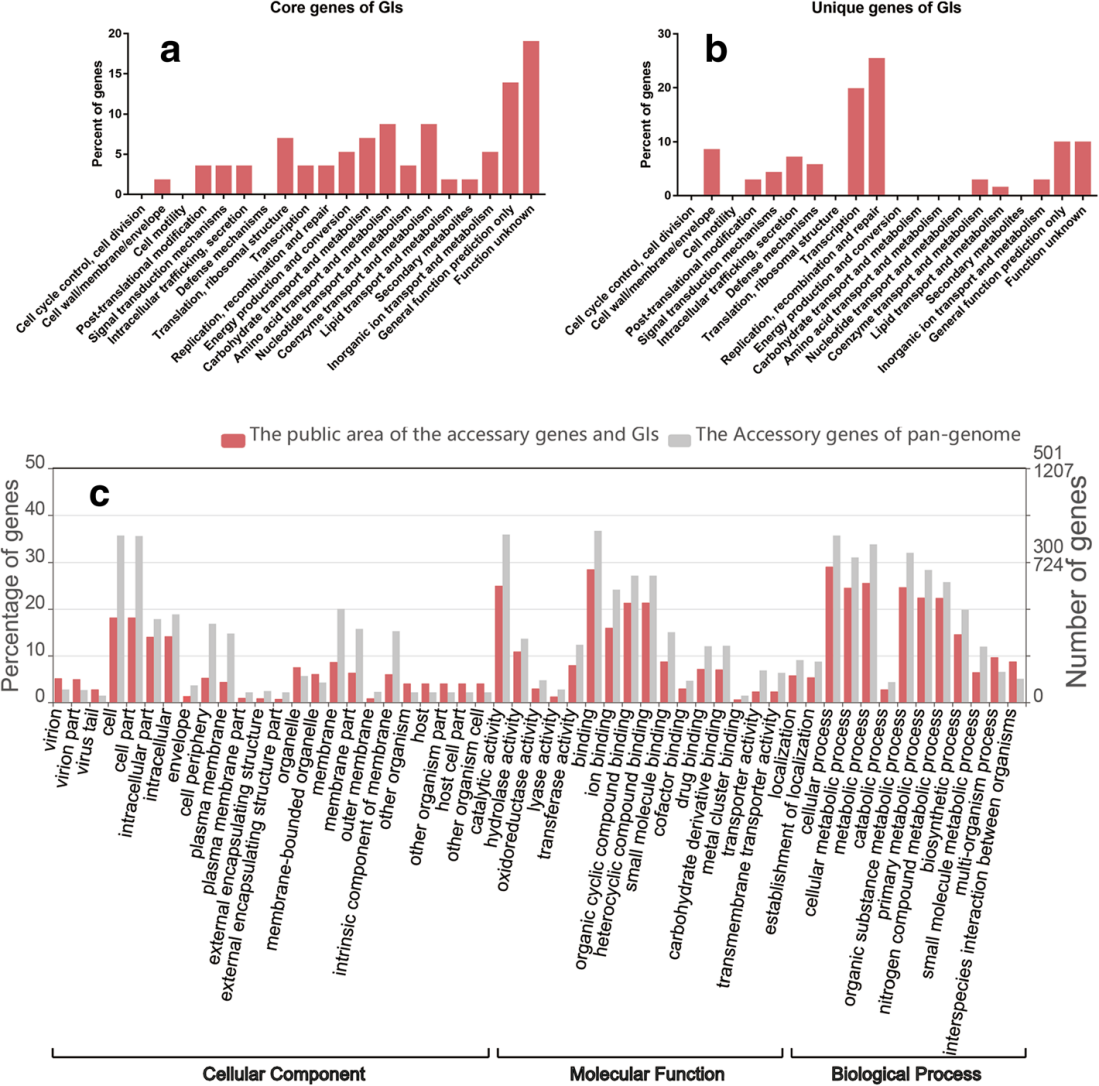
The functional properties of all the genes in the 280 GIs and their attribution in the pan-genome. The genomic sums of the 280 GIs predicted from 114 *P. multocida* strains are functionally clustered by the Cluster of Orthologous Groups (COG) database and Web Gene Ontology Annotation Plot (WEGO), respectively. **a** X-axis shows selected COG functional terms; y-axis shows the percentages of the core genes of GIs. **b** X-axis shows COG terms; y-axis shows the percentages of the unique genes of GIs. **c** X-axis shows selected GO terms; y-axis shows the percentages of the accessory GI genes

By comparing the core genes of the pan-genome of *P. multocida* with the genes contained in the GIs, approximately 5.8% (55) of the functional core genes were detected in the GIs. These genes existed in the non-gene island region of the same host strain many times and in the GIs region only at the two ends of the GI. According to COG function clustering, they were primarily associated with fundamental metabolic activities other than cell division and cell motility, and genes closely related to environmental adaptation and pathogenicity, including defence mechanisms, were not (Fig. 5a). In the accessory gene set, approximately 42.3% (1032) of the functional genes existed in the GIs. Similar to the main functional categories of the accessory gene set, the GI genes were compared with the general situation of the accessory gene set in the GIs by GO annotation and were mainly related to biological processes and cellular component (Fig. 5c), with enrichment associated with 97 functional genes (*p* > 0.05, FDR > 0.05) (Additional file 8: Table S5, Additional file 11: Table S6) of virion, membranes, extracellular region, symbiosis, and interspecies interaction. The unique genes in the strains were compared with those contained in GIs, and 200 of the 565 unique genes were found in the GIs, accounting for approximately 35.4% of the total. The functional proportions of all gene sets of the 280 GIs and the size regions occupied by the unique genes in each proportion were obtained (Fig. 5b). These genes were mainly related to the basic functional categories of replication and repair, but there were no major genes related to amino acid/nucleotide metabolism and ribosome structure, which are the most important for bacterial growth and reproduction; additionally, there were no genes related to secondary metabolism biosynthesis, but there was a proportion of genes related to defence mechanisms and cell membranes. Therefore, the unique genes mainly encoded proteins associated with symbiosis, virulence and resistance.

The prophage was an important source of GIs, and a significant element of GIs was the integrase/recombinase/transposase [6]. Of the predicted strain phage, 66 strains contained integrase/recombinase/transposase. Therefore, the phylogenetic tree based on the mobile elements in these prophage of 66 strains was used as a proxy for the phylogeny of the GIs. In the Additional file 12: Figure S5, the arc was marked as the same part compared to the phylogenetic tree based on the core genes, the green arc indicating the strains were in the same large branch, the black arc indicating them were scattered but all in a same branch, respectively. The phylogenetic relationship exhibited by the important mobile elements was basically consistent with the phylogenetic relationship of the core genes.

## Discussion

In the bioenergetics-constrained prokaryotic genome size, the adaptive evolutionary process of populations is revealed through the pan-genome [29, 30]. As all examples of genome reduction to date have been limited to endosymbionts or pathogens with a host-dependent lifestyle, we studied the open pan-genome of 114 *P. multocida* taxa (Fig. 1); our conclusions were similar to Hurtado et al [31] conclusions, indicating that this type of strain strengthens the ability of the “survival of the fittest” alone [32]. That is they are the pathogens of non-host dependent lifestyle from the open evolutionary pattern of *P. multocida*. The bacteria could expand their gene pool through a variety of mobile elements including GIs. HGT events with beneficial effects would increase the fitness of the acceptors, eventually being fixed in the population; whereas acquired genes being redundant or detrimental are subject to purifying selection, accumulating mutations before being pseudogeneticized or lost [33]. In the process, new genes with small fluctuations and the growing number of unique genes are the embodiment of the ability to obtain foreign elements stably and regularly (Fig. 2). However, differences in the gene levels of each strain might be due to geographical and ecological isolation, as shown in Fig. 4, where most of the similarly located *P. multocida* strains were in the same large branch, and most of the same host-derived were in the same phylogenetic tree. This contradicts Cao, et al. [20] who proposed phylogeny that showed no correlation with other species except for serotype. Moustafa, et al. [16] discovered that only the phylogeny of bovine strains was related to geographic location and host source. The phylogenetic tree of 114 strains based on core genes was compared with the phylogenetic tree of 146 *P. multocida* constructed by an alignment-free method with NCBI using feature (or l-mer) frequency profiles (FFP) of whole genomes [34]. As shown in Additional file 13: Figure S3, an arc of the same colour represents the same large branch. This comparison demonstrated that the loci of all strains in the phylogenetic tree had a high degree of similarity, except for the canis-sourced strain OH1905. OH1905 was not located on a single branch as on NCBI, but was located on the same large branch as P-1662 and some of the avian-source strains. This may be due to the different strains of the screening results making a slight change in common genes determined by these data, for example there are 306_PMUL, OH4807 in Additional file 13: Figure S3-A, while FDAARGOS_219 in Additional file 13: Figure S3-B. Genomic data for only one strain uploaded by a different research institution was retained in this paper, such as ATCC 11039 and X73, NCTC 10322 and ATCC 43137, and P-1059 and ATCC 15742. According to the MLST results (Additional file 14: Figure S4), OH1905 and P-1662 belong to two different MLST CC (clonal complexes), although both of them were unknown. This showed that they have a certain difference in gene level, so they should not be in the same branch but in line with their host source differences. Therefore, in this paper, the phylogeny of *P. multocida* accurately showed close relationships with geographical location and host origin.

Unquestionably, GIs are due to unique aspects of bacterial behaviour, and each of these important new genes brings a new possibility to this population [35]. As shown in Additional file 15: Table S8, the toxin HipA gene was closely related to the dormancy and persistence of bacteria, as well as an increase in antibiotic resistance; this gene was only present in the strain P1933 (WP_016533466.1) and the pathogenicity and resistance island GIPm3480_1 of the strains 3480 and GIPmHNO6_1 [36, 37]. The toxin HipA gene has been reported in *Escherichia coli* (*E. coli*), *Haemophilus parasuis* and is widely found in fungi but has not been reported in *Pasteurella* [38–40]. Simultaneously, GIPm3480_1 also possessed a gene for a CopG family transcriptional regulator that plays an antitoxin role similar to plasmid pEMB1 in *E. coli* [41]. GIPm3480_1 and multiple other strains contain the HNH endonuclease gene only carried by bacterium-infected COS phage. The HNH-TerS-TerL supramolecular complex in the GIs plus a complete capsid could cleave the cos-site of the phage. This is helpful to the island invasion packaging mechanism of the phage by certain helper phages to be excised, replicated, and packaged in an encapsulated form that can infect phage particles, thereby making GIs with no mobility inherent to their mobility [42, 43]. GIPmOH1905_1 and GIRazi_Pm0001_2 contained transcriptional regulator AlpA regulating multidrug/metal ion transporter genes and oxidative stress modulators [44]. In addition, GIPmHNO7_1 contained LPS-related proteins, iron uptake-related antibiotic biosynthesis monooxygenase, an anti-tetracycline gene, and a large number of ICE-related genes. GIPmHN06_3 contained dermonecrotic toxin. GIRazi_Pm0001_3 contained TonB-dependent receptors related to iron transport [45]. GIPm36950_1 contained an aminoglycoside antibiotic-resistance gene, aminoglycoside 3′-phosphotransferase, a beta-lactamase type resistance gene, a macrolide antibiotic resistance gene, tetracycline resistance genes, a nucleotidyl transferase gene, and multidrug efflux system-related proteins. It has been reported that this GI is ICEPmu1 with multiple drug resistance [23, 46]. According to statistics, there are multiple strains containing the ppGpp gene associated with starvation survival and virulence [47]. Xenobiotic response element (XRE) is a family transcriptional regulator involved in the stress response [48], and HicAB, a type II toxin-antitoxin system similar to *E. coli* HicAB, affects the growth and death of the strains [49]. The multitude of data and unique functions of GIs once again suggest this mobile element is likely related to the pathogenicity of strains and the host interactions in the process of population evolution [9].

Although the effect of HGT on prokaryotic evolution has been discovered [30], the precise interplay of bacterial GIs on population genomes is still poorly understood. Using *P. multocida* as an example, GIs accounted for 5.8% of the core genes in the pan-genome, 42.3% of the accessory genes, and 35.4% of the unique genes. The existence of a small number of core genes in the GI may be because these genes are not only widely present in *Pasteurella*, other types of bacteria, or even fungi. Therefore, when the entire GI is transferred to *P. multocida*, the genes with higher frequencies in the foreign strains were also transferred; however, because of their unique location characteristics at both ends of the GI, it is more likely that the host’s own genes have moved to the GI area after the GI was transferred to the host. Moreover, 42.3% of the accessory genes were derived from GIs. Most of the genes that have similar functional proportions to the accessory genes, possibly due to the greater demand for such functional categories during the survival and reproduction of these strains or the fact that most of the strains themselves contain many of the functional categories associated with biological processes to make GIs more likely to contain such genes. Gene enrichment of GIs within the accessory genes is an important concept, and these GI genes were fixed in the genome to enhance the invasiveness, anti-stress pathways and population genetic changes.

The unique genes were mostly exogenous, very likely to reflect the environmental pressure that the strains faced. As shown in Additional file 16: Table S9, there were 200 unique genes contributed by the GIs to strain 36,950, including most of the drug-resistant genes associated with the specific phenotype of the strain, such as aminoglycosides, macrolides, and beta-lactams. Most of the GI-related genes found in HS_SKN01 were related to ICE. The GI-related genes found in TX1 included many ICE-related proteins and genes involved in the metabolism of bacterial cell walls, ABC transporters, ATP-binding proteins containing both ATPase and permease components of an ABC-type multidrug transport system, cobalt transporters, membrane proteins, and TetR/AcrR family transcriptional regulator control genes involved in a variety of processes including antibiotic production, osmotic stress response, efflux pump expression, and multidrug resistance [50–52]. The unique genes of P-2095 located in the GI region included dTDP-4-dehydrorhamnose reductase associated with cell wall/membrane/envelope biogenesis [53]. Additionally, FDAARGOS_261 had the highest GC% (40.7%) of all strains and significantly higher number of unique genes than those of other strains (Additional file 2: Table S2), but most of these genes are hypothetical proteins. There were a number of hypothetical proteins with undefined functional categories besides FDAARGOS_261, so more research and data are needed to determine the uniqueness of these components.

In addition to the above unique parts of GIs for the population genome, the GIs might also have an impact on the population genetics of *P. multocida*. The matching of 63.3% prophage with the 17 complete GIs suggested the prophage was a possible mechanism of GIs. The phylogenetic relationship of GIs was demonstrated by the phylogenetic tree based on prophage (Additional file 14: Figure S4), in which 79% (49) strains exhibited the same phylogenetic relationship as the core genes. The remaining parts that did not show the same clustering results, but 18%(11) strains with close phylogenetic relationship (such as FDAARGOS_218 and ATCC15742, CIRMBP-0760 and CIRMBP-0758 etc.) were closely related to each other. Thus, the GIs might be correlated with the phylogeny of *P. multocida*, remained to be investigated more in detail. The availability of additional data about the strains could pave the way to quantitative insights into the evolution of these bacteria.

We demonstrated that the pan-genome components of *P. multocida* were disparately affected by GIs, which played an important role in the amplification of the pan-genome of bacteria flora, especially the genes related to virulence, tolerability and environmental adaptability. These types of genes enable the bacteria to transfer benefits to themselves through the horizontal GI transfer, thereby enhancing symbiosis and adaptation of the bacteria.

## Conclusion

Through pan-genome analysis, the phylogeny of *P. multocida* was found to be closely related to host source and geographical location. The GI genes found in the population genome of *P. multocida* were scrutinized. We identified 280 GIs, accounting for 5.8% of the core genes in the pan-genome, 42.3% of the accessory genes and 35.4% of the unique genes. The identified genes were mainly related to strain virulence and environmental adaptation. Therefore, in the context of population genetics of *P. multocida*, further studies analysing the contribution of GIs to strain adaptation and evolutionary benefit, followed by a large quantity of GI data analysis in population genome will lead to a more diverse understanding of strain adaptation and evolution.

## Materials and methods

### Source of genome sequences

Three isolates were identified as *P. multocida*, namely DY120818, HN141014 and RCAD0276, from China that originated from dead duck livers were utilized, as well as six avian origin *P. multocida* strains obtained from the American Type Culture Collection (ATCC) and one *P. multocida* CVCC44801 (C48) obtained from the China Veterinary Culture Collection Center (CVCC). Sequencing of the 10 isolates was conducted using an Illumina HiSeq 2500. High-quality genomic DNA was extracted with a TIANamp Bacteria DNA Kit (Tiangen Biotech Co, Ltd., Beijing, China). Illumina reads were corrected by Quake [54]. De Novo assembly was performed using two different assembly tools: Velvet v1.2.09 [55] together with VelvetOptimiser [56]. The VelvetOptimiser was used to optimize assembly parameters automatically, and SPAdes version 3.1.0 [57] was used with default parameters. The other 104 *P. multocida* complete genome sequences were retrieved from GenBank at NCBI [58]. The genome completeness of all strains was evaluated using the CheckM14 genome quality estimator [59].

### Calculation of pan-genome size and gene sets

BPGA, a fast pan-genome analysis tool, was used to analyse the related genomes of all strains [60]. The cut-off of Blast is 50% without paralogous clustering. The median sizes of the pan-genomes data were generated using the mathematical model heap’s law, n_pan_ = k_pan_N^γ^ [25]. The N is the number of genomes, both k_pan_ and γ are free parameters, and n_pan_ is the total number of all non-redundant gene families in the pan-genome. We retrieved the orthologous genes of various genomes and their classifications as core genes, accessory genes, unique genes and new genes.

### MLST analysis

The sequence typing (ST) and clonal complexes (CCs) of 114 *P. multocida* were determined by means of BioNumerics software resorting a temporary evaluation license from Applied Maths [61]. In Multi Locus Sequence Typing (MLST), an ST is uniquely determined by the allelic profiles, and CCs differing at one of the seven MLST loci are grouped into STs that share a recent common ancestor [62].

### The construction of the phylogenetic tree

The nucleotide sequences corresponding to the core genes of 114 strains were concatenated to construct a core genome concatamer. The core genome concatamer was aligned using the MAFFT online service [63] (default parameters) and then a neighbour-joining (NJ) trees was constructed using MEGA programme on the basis of the Jensen-Shannon divergences matrix from each type of genome partition [64]. The p-distance of the tree based on core genes was calculated as 0.020, and the average Jukes-Canter (JC) distance was 0.009. Branch support was computed by bootstrap with 1000 replicates, and “Maximum Composite Likelihood” model was used to construct the resulting graph. The MLST sequence was analysed in the same way with a p-distance of 0.010 and a JC distance of less than 1, with 1000 bootstrapping replications. And the phylogenetic tree based on integrase/recombinase/transposase of prophage was constructed in the same way. Finally, the tree was recomputed to determine the disproportionation branches with > 50 uncertainty, and these branches were placed in the same larger branch and converted into a consistent tree in the graph.

### Identification of genomic islands

The whole genome sequences across all strains were upload to IslandViewer4 [65], where the potential GI regions were predicted by at least one method and expounded explicitly. All data for the 114 taxa were reinterpreted with the Kyoto Encyclopedia of Genes and Genomes (KEGG) [66] and Cluster of Orthologous Groups (COG) [67]. The approximate multi-collinearity of 17 complete genomes of *P. multocida* were compared to each other by means of Mauve, which was utilized to analyse transmutation and development among strains such as inversion, rearrangement, insertion and deletion in the shape of locally collinear blocks (LCBs) [68, 69]. The existing GIs were preliminarily screened on the basis of the unique or minimal number of comparative genomic characteristics of GI by Mauve [70]. The hypothetical protein data output was validated again by BLAST and the Conserved Domains Database (CDD) [71] of NCBI to further determine the functional categories. Furthermore, the 17 obtained GIs demonstrated precise locations according to the various characteristics, including common GI themes, abnormal GC content, direct repeat (DR) structure flanking, several genes encoding traits that may increase bacterial adaptability or fitness under certain growth conditions, fragmented insertion sequence (IS) elements and other mobility-related genes (e.g., essential functional integrase (Int), recombinase or transposase); the functions of these genes are involved in the insertion and deletion of the region that is flanked by DR structures and are absent from the chromosome with which they are closely associated [6, 72]. The detailed information of the GIs of 114 strains as Additional file 6: File S1 could be seen in https://figshare.com/s/405c87082feea840e2da (DOI: 10.6084/m9.figshare.7440329).

### Calculation of intersection between GI and pan-genome

These genes present on the GIs in the core genes, the accessory genes and unique genes. The common part of the core genes and GIs were analysed by the COG database for their functional clustering results, and the common parts of the unique genes and GIs were the same. In the background of the newest (2018-03-01) gene ontology files, the shared parts of the accessory genes and the GIs were used to obtain the gene enrichment at the 3rd level of GO in the native format with the Web Gene Ontology Annotation Plot (WEGO) [73].

### Identification of prophage regions

Bacteriophages (phages) are bacterial viruses that infect bacteria, fungi, and actinomyces, while the prophage is the nucleic acid of a mild phage which is integrated into the host genome [74]. According to PHASTER (PHAge Search Tool Enhanced Release) [75], a web of online analysis using the previously retrieved genome sequences, prophage sequences within all bacterial genomes were identified, annotated and forecasted successfully. The detailed information of the predicted prophage of 114 strains as Additional file 9: File S2 could be seen in https://figshare.com/s/405c87082feea840e2da (DOI: 10.6084/m9.figshare.7440329).

## Additional files

Additional file 1: **Table S1.** The origin and genomic characteristic of 114 *P. multocida*. (XLSX 26 kb)

Additional file 2: **Table S2.** The General features of 114 strains genome. (XLSX 17 kb)

Additional file 3: **Figure S1.** The ratio of pan-genome in each strain. (TIF 2830 kb)

Additional file 4: **Table S3.** The pan-genome detailed statistical data. (XLSX 13 kb)

Additional file 5: **Figure S2.** The phylogenetic tree of 114 *P. multocida* based on MLST. (TIF 1599 kb)

Additional file 6: **File S1.** Detailed information on GI of each strain (ZIP 1360 kb).

Additional file 7: **Table S4.** Specific information of the GIs on 17 complete genome. (XLSX 14 kb)

Additional file 8: **Table S5.** A summary of the number of the GIs of 97 whole genome. (XLSX 14 kb)

Additional file 9: **File S2.** Detailed information on predicted phage of each strain (ZIP 5010 kb).

Additional file 10: **Table S7.** Predicted phage basic information. (XLSX 79 kb)

Additional file 11: **Table S6.** Enriched genes of GIs in the accessory gene. (XLS 1835 kb)

Additional file 12: **Figure S5.** The phylogenetic tree based on prophage. (TIF 1439 kb)

Additional file 13: **Figure S3.** Comparison of the phylogenetic tree 147 Pm from NCBI. (TIF 5215 kb)

Additional file 14: **Figure S4.** The likelihood tree of 114 *P. mltocida* based on MLST. (TIF 347 kb)

Additional file 15: **Table S8.** The significant functional genes in GIs of *P. multocida*. (XLSX 12 kb)

Additional file 16: **Table S9.** The significant functional genes in the common part of the GIs and the unique genes of the pan-genome. (XLSX 10 kb)

## Abbreviations

AR: Atrophic Rhinitis
ATCC: American Type Culture Collection
CCs: Clonal Complex
CDD: Conserved Domains Database
CDSs: Coding DNA Sequences
COG: Cluster of Orthologous Groups
CVCC: China Veterinary Culture Collection Center
DR: direct repeat
FC: Fowl Cholera
GIs: Genomic Islands
HGT: horizontal gene transfer
HS: Haemorrhagic Septicaemia
ICE: Integrative and Conjugative Elements
Int: integrase
IS: insertion sequence
KEGG: Kyoto Encyclopedia of Genes and Genomes
LCB: Locally collinear block
MLST: Multi Locus Sequence Typing
NCBI: National Center for Biotechnology Information
PHASTER: PHAge Search Tool Enhanced Release
ST: Sequence Typing
WEGO: Web Gene Ontology Annotation Plot
