# Supplementary figures and images for: Comparative analysis reveals the Genomic Islands in Pasteurella multocida population genetics: on Symbiosis and adaptability
Source: BMC Genomics. 2019 Jan 18;20:63. doi: 10.1186/s12864-018-5366-6 (PMC6339346; doi:10.1186/s12864-018-5366-6)

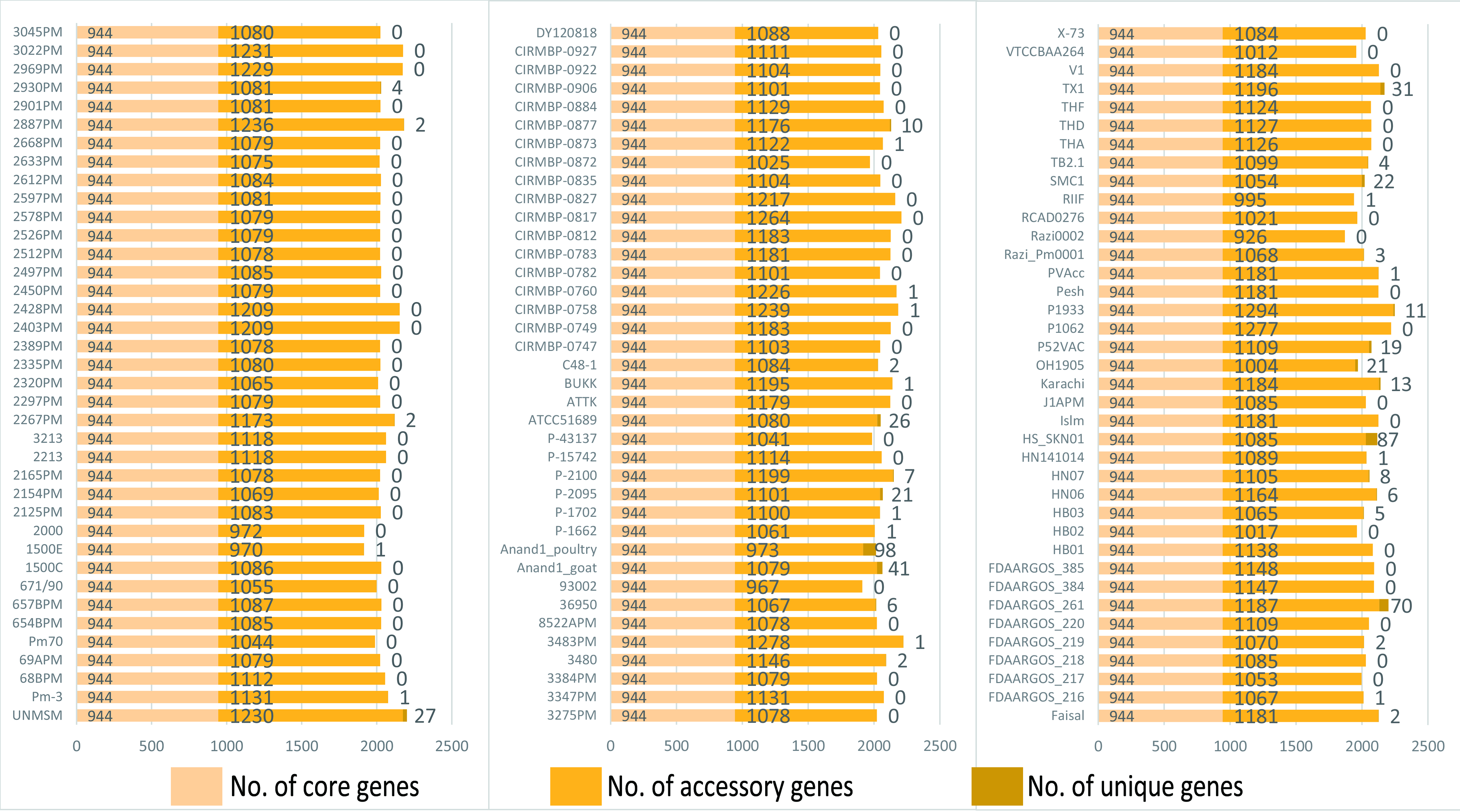

Supplement: Supplementary file 3 — Figure S1. The ratio of pan-genome in each strain. (TIF 2830 kb) [file 12864_2018_5366_MOESM3_ESM.tif]

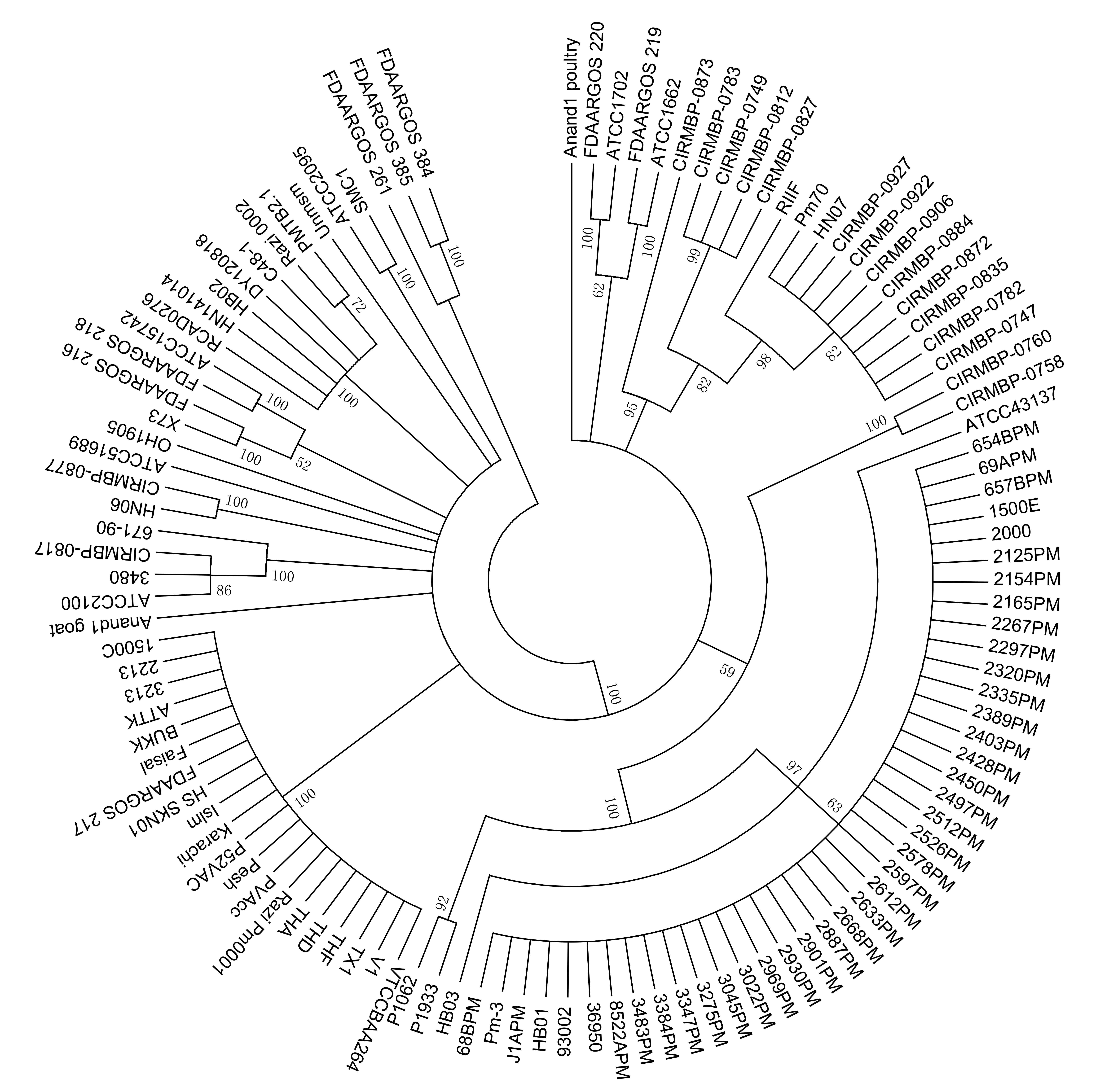

Supplement: Supplementary file 5 — Figure S2. The phylogenetic tree of 114 P. multocida based on MLST. (TIF 1599 kb) [file 12864_2018_5366_MOESM5_ESM.tif]

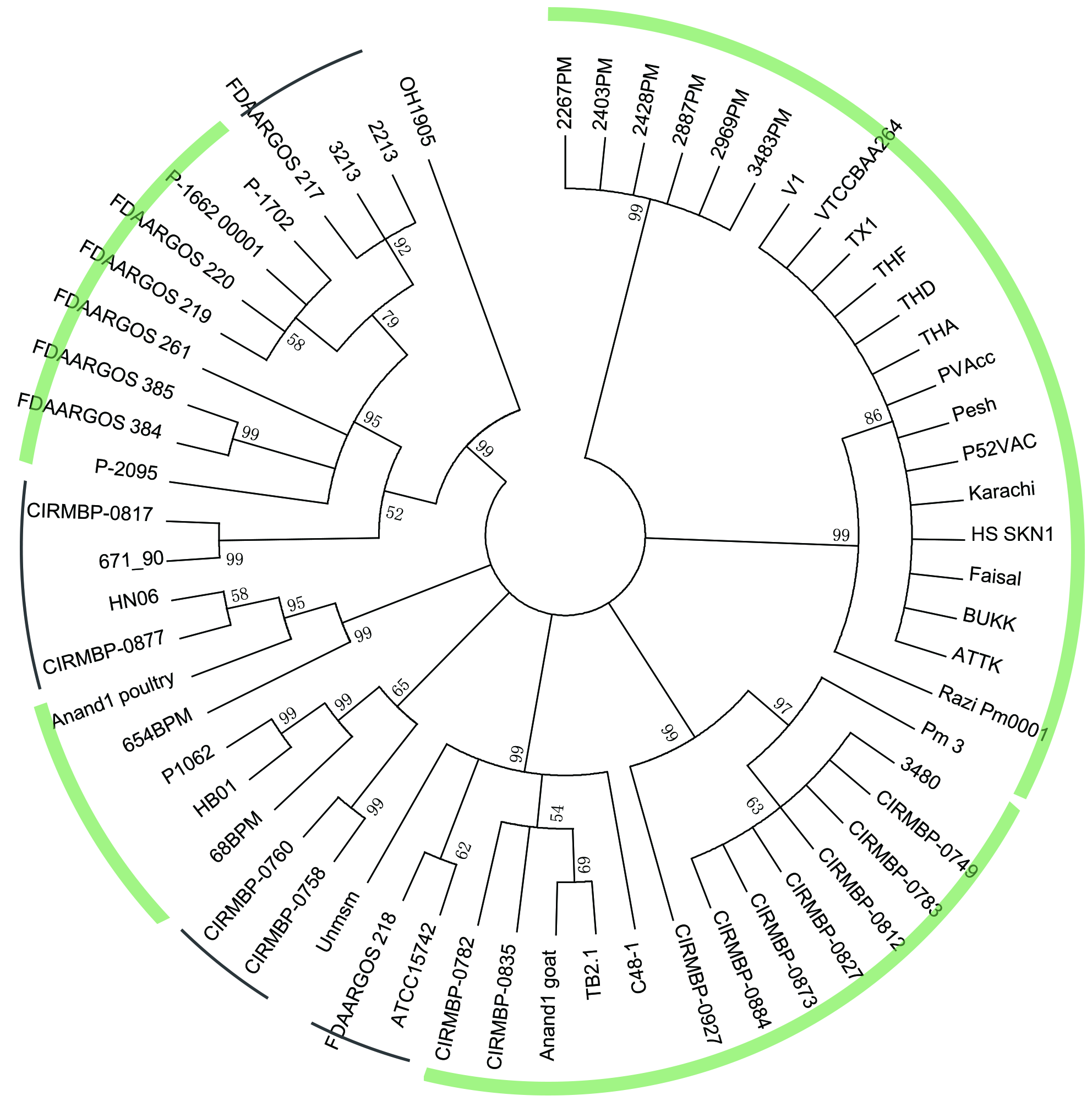

Supplement: Supplementary file 12 — Figure S5. The phylogenetic tree based on prophage. (TIF 1439 kb) [file 12864_2018_5366_MOESM12_ESM.tif]

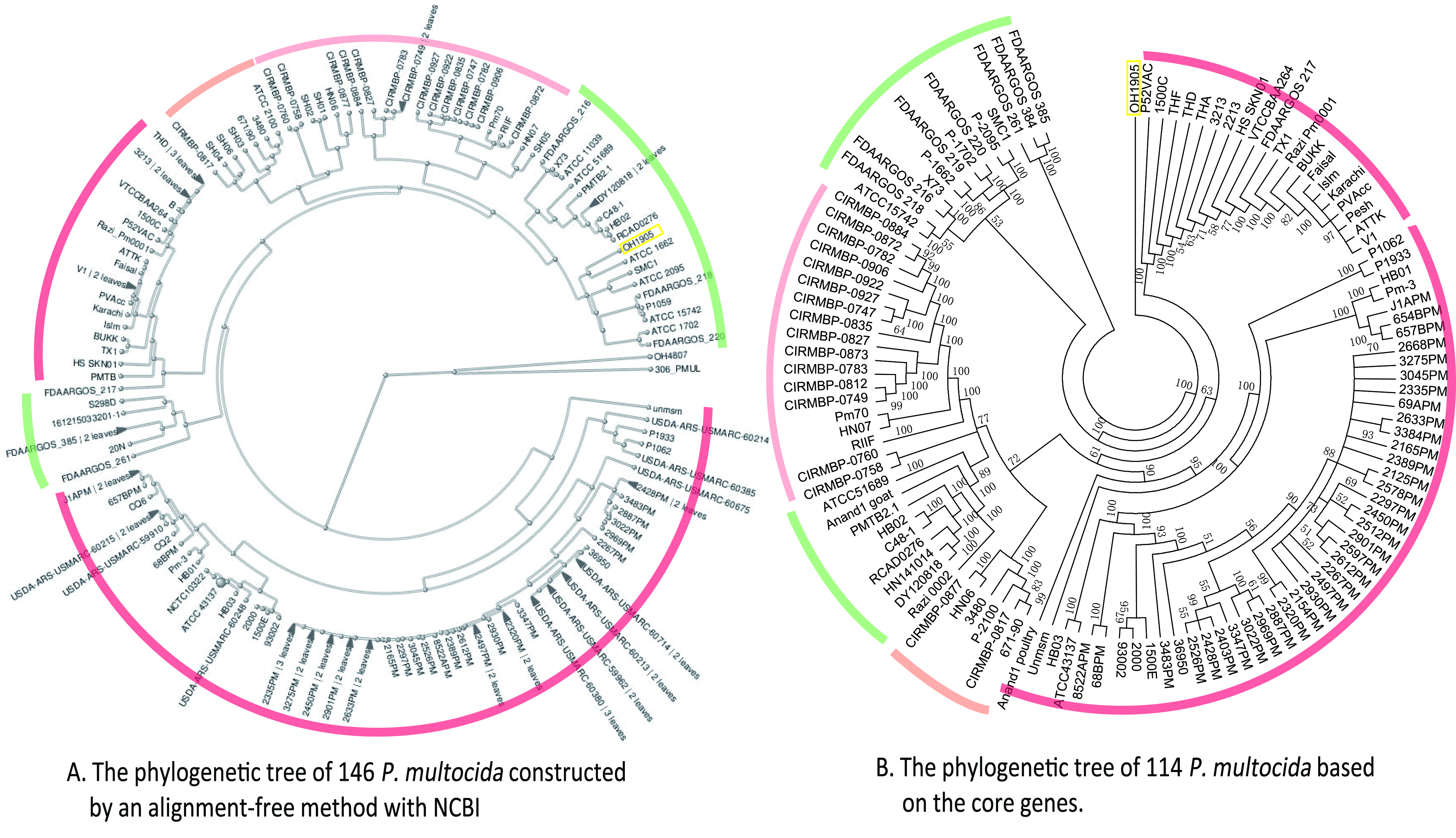

Supplement: Supplementary file 13 — Figure S3. Comparison of the phylogenetic tree 147 Pm from NCBI. (TIF 5215 kb) [file 12864_2018_5366_MOESM13_ESM.tif]

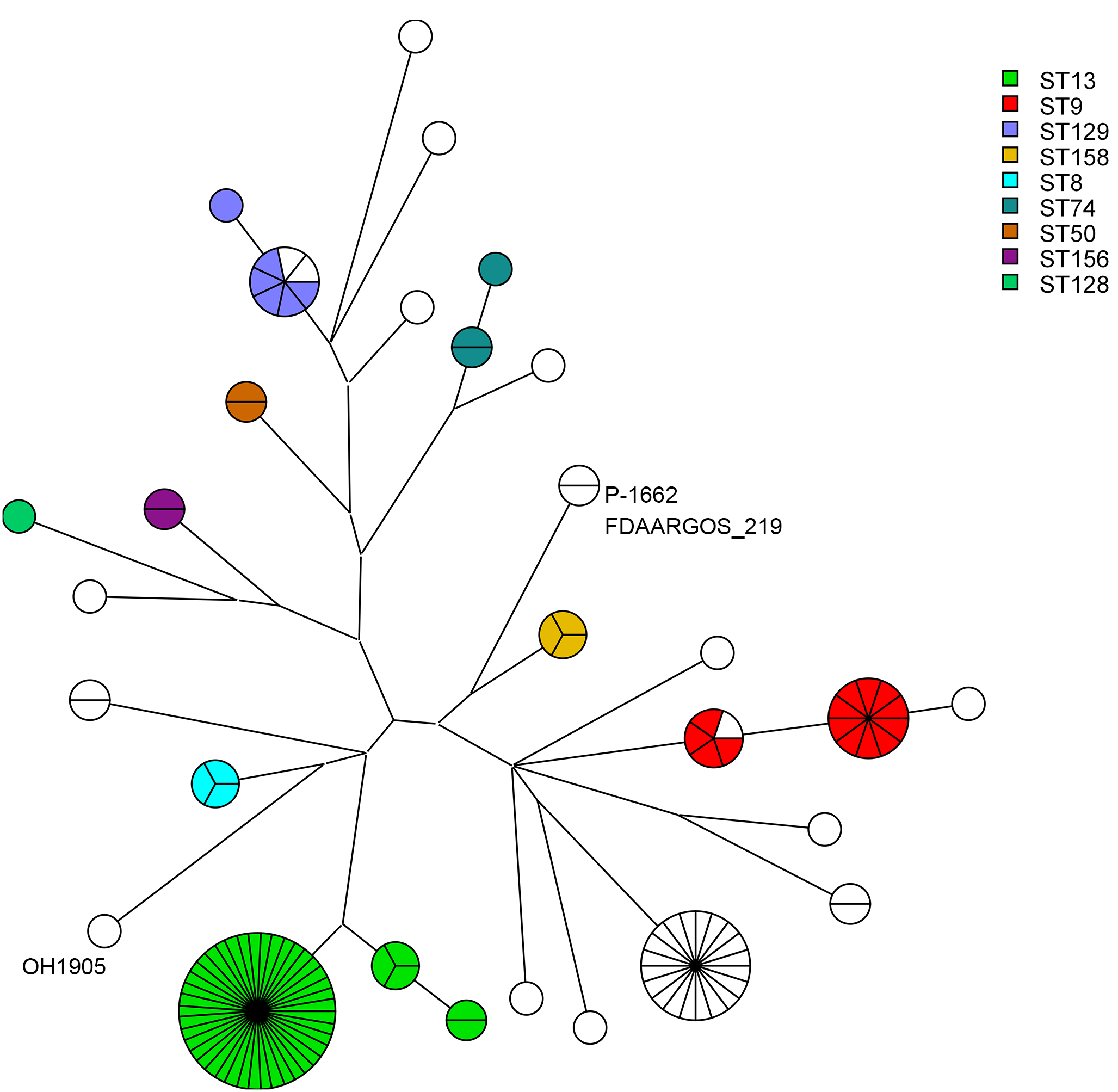

Supplement: Supplementary file 14 — Figure S4. The likelihood tree of 114 P. mltocida based on MLST. (TIF 347 kb) [file 12864_2018_5366_MOESM14_ESM.tif]
